# Chronic olanzapine administration causes metabolic syndrome through inflammatory cytokines in rodent models of insulin resistance

**DOI:** 10.1038/s41598-018-36930-y

**Published:** 2019-02-07

**Authors:** Huqun Li, Shiyong Peng, Shihong Li, Shouqing Liu, Yifan Lv, Ni Yang, Liangyu Yu, Ya-Hui Deng, Zhongjian Zhang, Maosheng Fang, Yunxiang Huo, Ying Chen, Taohua Sun, Weiyong Li

**Affiliations:** 1 Department of Pharmacy, Union Hospital, Tongji Medical College, Huazhong University of Science and Technology, Wuhan, 43000 P. R. China; 2 Institute of Psychiatry and Neuroscience, Xin Xiang Medical University, Xinxiang, Henan 435000 China; 3 Section on Developmental Genetics, PDEGEN, NICHD, Bethesda, Maryland 20892 USA; 4 Wuhan Mental Health Center, Wuhan, 430019 P. R. China; 5 Wuhan Youfu Hospital, Wuhan, 430050 P. R. China; 6 Qingdao Municipal Hospital, Qingdao, 266011 P. R. China

## Abstract

Olanzapine is a second-generation anti-psychotic drug used to prevent neuroinflammation in patients with schizophrenia. However, the long-term administration of olanzapine leads to insulin resistance (IR); the mechanisms of this effect remains poorly understood. Using cellular and rodent models of IR induced by olanzapine, we found that chronic olanzapine treatment induces differential inflammatory cytokine reactions in peripheral adipose and the central nervous system. Long-term treatment of olanzapine caused metabolic symptoms, including IR, by markedly elevating the plasma levels of pro-inflammatory cytokines, including IL-1ß, IL-6, IL-8 and TNFα; these findings are consistent with observations from schizophrenia patients chronically treated with olanzapine. Our observations of differential inflammatory cytokine responses in white adipose tissues from the prefrontal cortex in the brain indicated cell type-specific effects of the drug. These cytokines induced IR by activating NF-kB through the suppression of IkBα. Functional blockade of the components p50/p65 of NF-kB rescued olanzapine-induced IR in NIH-3T3 L1-derived adipocytes. Our findings demonstrate that olanzapine induces inflammatory cytokine reactions in peripheral tissues without adversely affecting the central nervous system and suggest that chronic olanzapine treatment of schizophrenia patients may cause inflammation-mediated IR with minimal or no adverse effects in the brain.

## Introduction

Schizophrenia is a common mental illness that causes obstacles in thinking, emotion and behavior and is characterized by incongruity between mental activities and the environment^1,2^. This severe psychosis has unknown causes with more than 1% incidence^3^ and has a lifelong impact on patients from its onset in young adulthood^4,5^. Schizophrenia seriously impacts patients’ cognitive, daily life and social functions, which impose many problems and burdens on patients’ families, community and society^6^. Schizophrenia was misunderstood for centuries, and there was no modern treatment for schizophrenia until the 1950s^7^.

The second generation antipsychotics (SGAs) are not limited to the blocking of dopamine D2 receptors; instead, they have a dual blocking effect on the dopamine D2 receptor and 5-hydroxytryptamine-2 (5HT2)^3,8^. Olanzapine, one of the most widely used SGAs, is effective for both the positive and negative symptoms of schizophrenia. Since 1996, olanzapine has effectively improved the qualities of patients’ lives and the mental aspects of the pathology with only mild to moderate sedation^9,10^. Due to its higher clinical efficacy and decreased EPS, olanzapine has been globally used in the treatment of other mental illnesses, such as bipolar disorder and major depression disorders (MDD)^8,11,12^.

Recent clinical studies and reviews have indicated that chronic olanzapine treatment is often associated with severe metabolic side effects, such as obesity, dyslipidemia and insulin resistance (IR), which increase the risk of type 2 diabetes, cardiovascular disease and noncompliance in schizophrenia patients^13–19^. However, the mechanisms that underlie chronic olanzapine-induced IR remain debatable, which, in turn, largely restrict olanzapine as a maintenance treatment against severe schizophrenia^20,21^.

Studies suggest that olanzapine binds with the neurotransmitter receptors H1 and M3 in the hypothalamus, which increases food intake and body weight leading to IR^22–25^. However, more recent clinical observations have indicated that chronic olanzapine treatment caused metabolic abnormalities and IR without weight gain^26–29^. These studies found that these receptors were also abundantly distributed in peripheral tissues, such as the pancreas and liver. It was stated that the acute administration of olanzapine could induce IR through increasing hepatic glucose production, decreasing glucose uptake, and rapidly inducing adverse metabolic reactions even before weight gain occurred.

Recent studies have proposed IR as an inflammatory disease^30^. Inflammation could activate serine but not threonine phosphorylation of the insulin receptor substrate (IRS), prevent PI3K activity and impair insulin signal transduction via dissociation of IRS-PI3K formation^31,32^. Consequently, serine phosphorylation could inhibit the translocation of glucose transporter 4(GLUT-4) to the plasma membrane and the uptake of glucose into adipocytes and muscle resulting in IR^33^. Numerous clinical and animal studies have shown that brain inflammation is closely related to schizophrenia pathophysiology^34–37^. More interestingly, olanzapine could prevent inflammatory mediation by reducing glial activity in primary glia cells cultured from rat newborn brains^38,39^. However, female rats that received olanzapine treatment had elevated plasma levels of IL-8 and IL-1β and increased macrophage infiltration in white adipose tissue in the peripheral system. Given that olanzapine is reported to induce the production and release of inflammatory factors from peripheral adipose tissue, we hypothesized that long-term olanzapine treatment induces IR by activating the inflammatory cytokine response in these tissues.

In the present study, proinflammatory factors were investigated in schizophrenia patients with long-term olanzapine treatment in different psychiatric hospitals. We then further explored the molecular mechanisms of olanzapine induced IR using peripheral adipose from animal models and cellular culture systems. Our results demonstrated the relationship between inflammatory cytokines and insulin resistance in clinical patients, olanzapine-treated mice and adipocytes. Moreover, our results demonstrated that long-term olanzapine application did not induce an inflammatory reaction in the central nervous system; however, it caused IR in adipose tissue via activation of the NF-κB inflammation cascades. The current study proposed new mechanisms underlying olanzapine induced IR and may provide alternative ways of modifying olanzapine therapy for schizophrenia patients.

## Results

### Schizophrenia Patients Developed IR after Long-term Olanzapine Maintenance Treatments

Clinical patients from different hospitals were enrolled in this study. The body weight, fasting blood glucose and insulin levels were measured, and the insulin resistance index was calculated. To fulfill our investigation purpose, we selected 14 patients with less or no insulin index change compared to the average levels in the normal subjects, grouped as OS, and 14 additional patients with a significantly higher insulin index, referred to as OR (Table 1). Compared with OS, the patients in the OR group had significantly increased body weight, fasting blood glucose and insulin levels, and the HOMA-IR significantly increased, which indicated an insulin resistance status (Table 1).

**Table 1:** Demographic and clinical characteristics of subjects.

Characteristics	OS^&^ group	OR^#^ group
Patients (N)	14	14
Age (yr)	35.3 ± 10.4	33.8 ± 7.9
Gender (m/f)	7/7	8/6
Weight (kg)	62.5 ± 11.3	75.6 ± 14.6*
Fasting insulin (μU/mL)	5.5 ± 1.0	7.6 ± 1.6*
Fasting glucose (mmol/L)	4.7 ± 0.4	5.1 ± 0.5*
HOMA-IR index	1.2 ± 0.2	1.7 ± 0.4*

^&^OS indicates olanzapine sensitive group; ^#^OR indicates olanzapine resistance group. HOMA-IR index = fasting blood glucose (mmol/L) *fasting insulin (mU/L)/22.5. *p < 0.05 in OR group compared with patients in OS group.

**Figure 1:**
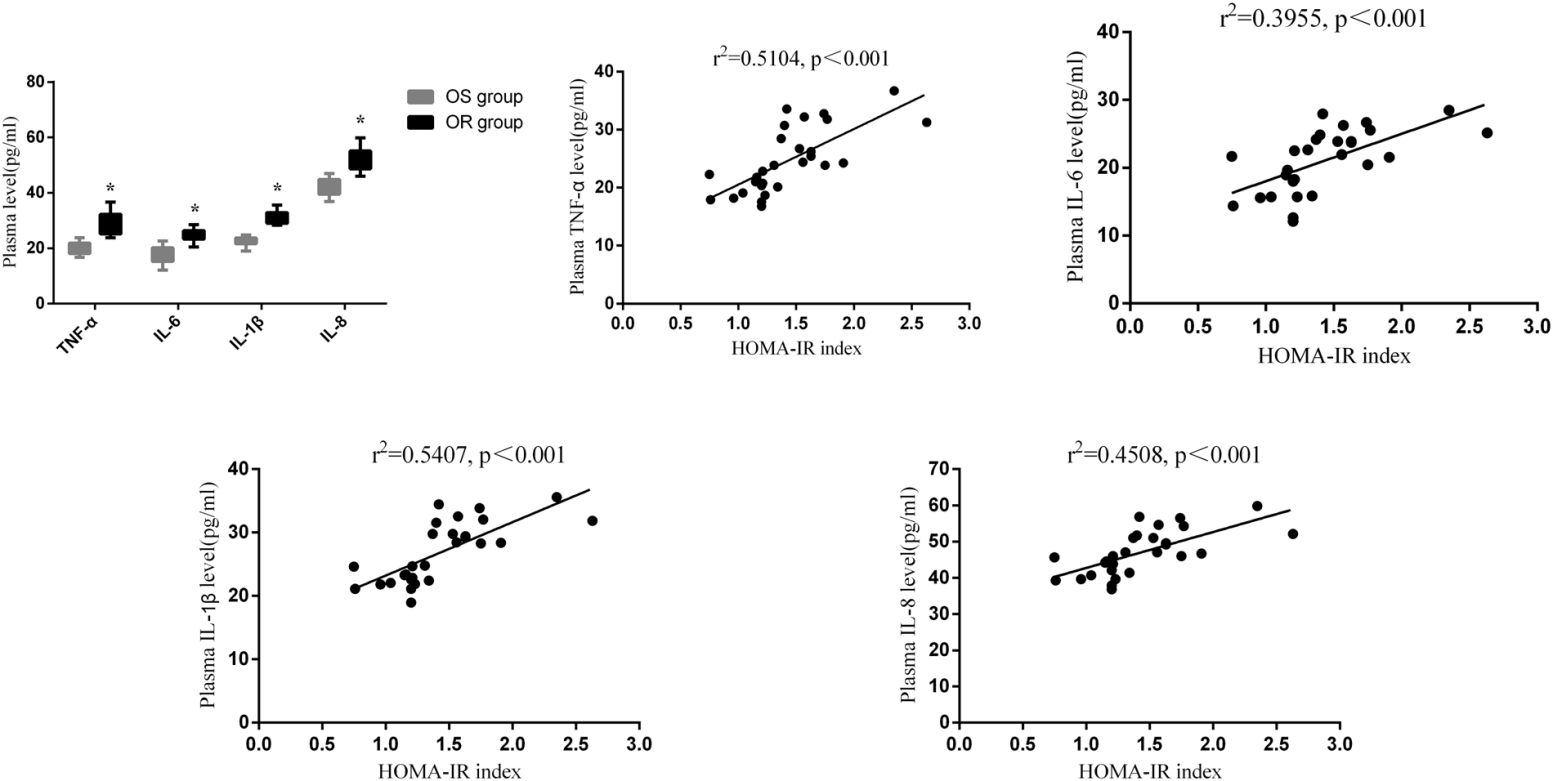
Insulin resistance in patients increased along with levels of plasma inflammatory cytokines. Inflammatory cytokines were correlated to insulin resistance indexes. (**A**) Plasma TNF-α, IL-6, IL-1β and IL-8 levels in patients. (**B**) Correlation between TNF-α and HOMA-IR index. (**C**) Correlation between IL-6 and HOMA-IR index. (**D**) Correlation between IL-1β and HOMA-IR index. (**E**) Correlation between IL-8 and HOMA-IR index. OS, olanzapine sensitive; OR, olanzapine resistance. n = 14 per group. Student *t-test* was used for comparisons between OS and OR groups. The Pearson correlation coefficient scores were calculated among plasma TNF-α, IL-6, IL-1β and IL-8 levels and HOMA-IR index. Data are presented as mean ± SD. *p < 0.05.

To determine whether inflammation is closely related to insulin resistance in OR patients, we collected plasma after long-term olanzapine treatments from both groups. ELISA experiments showed that the plasma levels of TNF-α, IL-6, IL-1β and IL-8 in the OR group were significantly higher than those in the OS group (Fig. 1A, p < 0.05). Linear regression statistical analyses demonstrated that the HOMA-IR index changes were closely correlated with these inflammatory factors (Supplemental Table 2), including TNF-α (Fig. 1B, r^2^ = 0.5104, p < 0.001), IL-6 (Fig. 1C, r^2^ = 0.3995, p < 0.001), IL-1β (Fig. 1D, r^2^ = 0.5407, p < 0.001), and IL-8 (Fig. 1E, r^2^ = 0.4508, p < 0.001).

### Rodent Models with Chronic Olanzapine Treatment Mimic Inflammatory Reactions in Peripheral System but not in Central Nervous System

To further confirm whether long-term application of olanzapine induces inflammation, we tested this hypothesis in mouse and rat models. Following intraperitoneal injection of olanzapine, 10 mg/kg for 8 weeks, the fasting blood glucose and insulin were measured, and the IR index was calculated. Compared with the control group (CG group in mouse or BL in rat), the mice in the olanzapine treatment group (OL group in mice) did not exhibit a significant increase in the body weight; however, the rats in the olanzapine treatment (IR group in rats) had a significant increase in the body weight (Supplemental Table 1). The levels of fasting blood glucose and insulin and the IR index significantly increased for both the OL and IR groups. In the oral glucose tolerance test (OGTT analysis), the area under the blood glucose curve was significantly increased in the OL or IR group compared with the control group (Supplemental Table 1).

**Figure 2:**
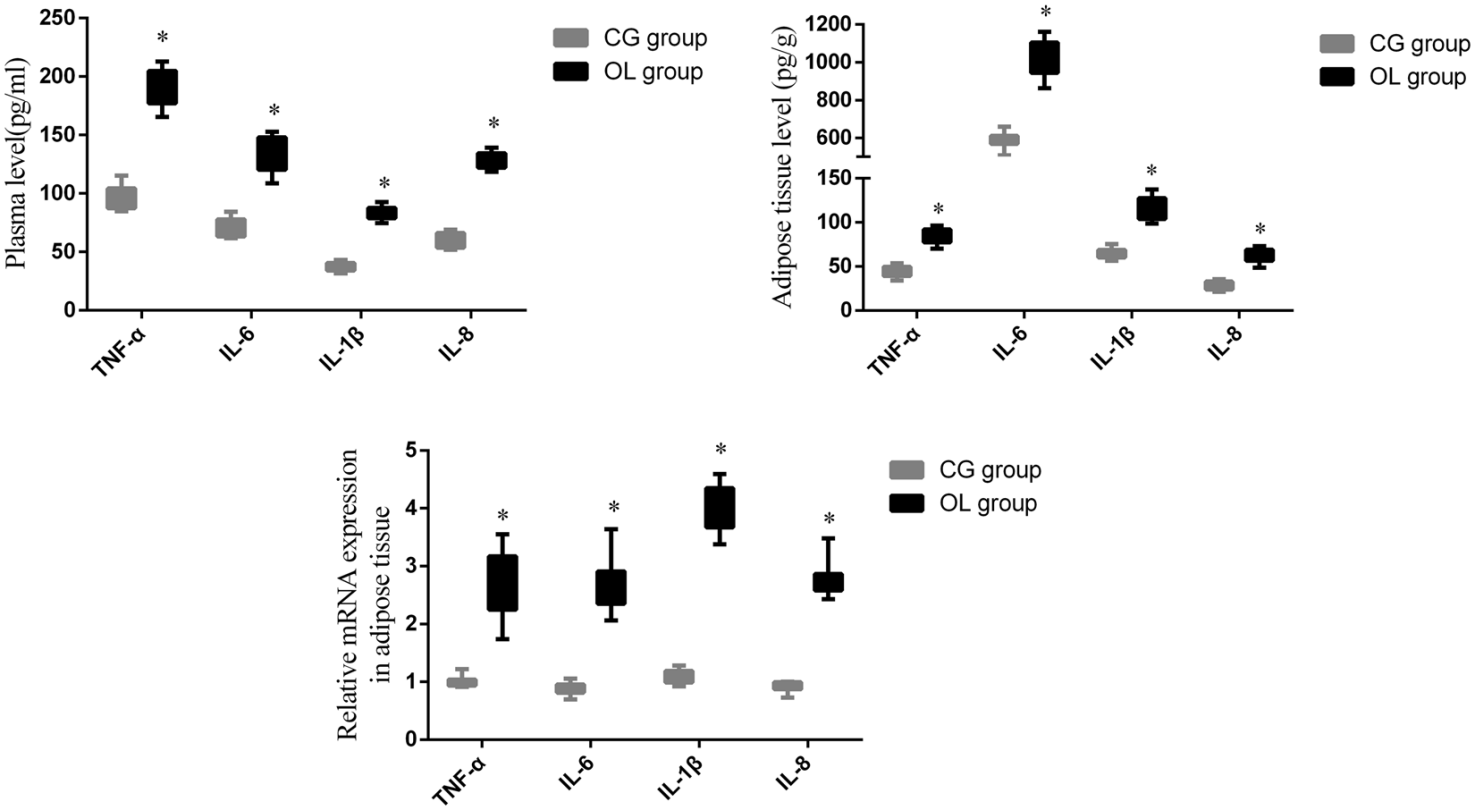
Chronic olanzapine treatment altered inflammatory cytokines in mice. Inflammatory cytokines were correlated to IR. (**A**) Plasma levels of TNF-α, IL-6, IL-8 and IL-1β in mice. (**B**) Levels of TNF-α, IL-6, IL-8 and IL-1β in adipose tissue in mice. (**C**) Relative mRNA expression of TNF-α, IL-6, IL-8 and IL-1β in adipose tissue in mice. Student *t-test* was used for comparisons of inflammatory cytokines between mouse groups. Data are presented as mean ± SD. *p < 0.05.

The levels of TNF-α, IL-6, IL-8 and IL-1β in the plasma and adipose tissue of the mice were detected by ELISA. The expressions of TNF-α, IL-6, IL-8 and IL-1β mRNA in the adipose tissue were detected by quantitative real-time PCR. Compared with the CG group, the plasma levels of TNF-α, IL-6, IL-1β and IL-8 in the OL group significantly increased (Fig. 2A, P < 0.05). The TNF-α, IL-6, IL-1β and IL-8 in the adipose tissue were also significantly increased (Fig. 2B, p < 0.05). Compared with the CG group, the expressions of TNF-α, IL-6, IL-8 and IL-1β mRNA in the adipose tissue of the OL group were also significantly increased (Fig. 2C, p < 0.05). Moreover, Pearson’s correlation analysis showed that all inflammatory factors in the plasma were correlated with the serum insulin levels (Supplemental Fig. 1a–d and Supplemental Table 3.).

**Figure 3:**
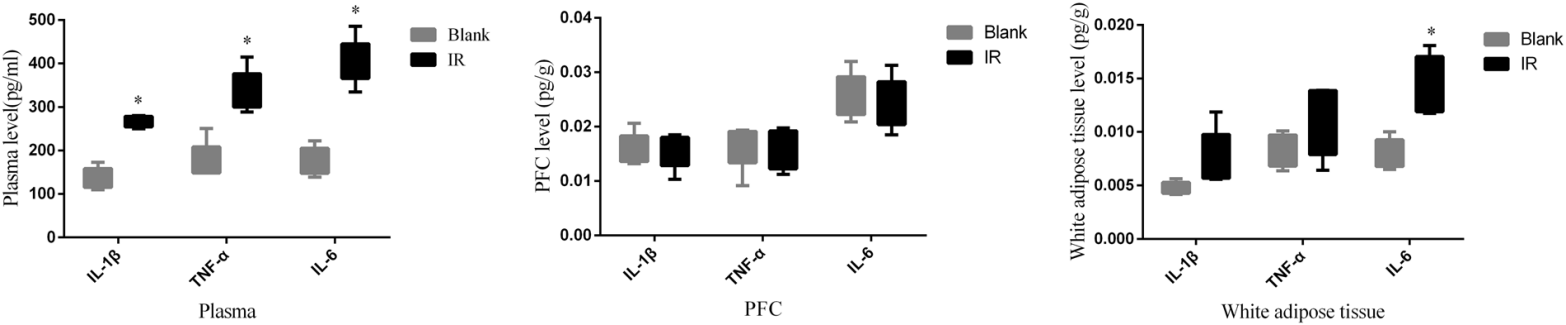
Chronic olanzapine treatment significantly triggered release of inflammatory cytokines in rat adipose tissue, but did not induce inflammation reactions in brains. (**A**) Levels (pg/ml) of TNF-α, IL-6 and IL-1β in rat plasma were detected using ELISA. (**B**) levels (pg/g) of TNF-α, IL-6 and IL-1β in rat white adipose tissue were tested using ELISA. (**C**) Levels (pg/g) of TNF-α, IL-6 and IL-1β in rat prefrontal cortex tissue (PFC) using ELISA. Student t-test was used for comparisons of inflammatory cytokines between mouse groups. Data are presented as mean ± SD. *p < 0.05.

Brain inflammation is closely related to schizophrenia pathophysiology, and olanzapine could prevent the neuroinflammation of schizophrenia brains in clinical studies^32^. Thus, we hypothesized that olanzapine may have distinct activities in peripheral adipose and central nervous systems. We detected several major inflammatory factors in chronic olanzapine-treated rat models. Compared to the age-matched control group, the ELISA quantitative data demonstrated that olanzapine could induce significant increases in inflammatory cytokines, including TNF-α, IL-6, and IL-1β, in the plasma and white adipose tissues (Fig. 3A–C). Interestingly, there were no differences in the levels of TNF-α, IL-6, and IL-1β in the prefrontal cortex (PFC) between the olanzapine-treated and age-matched rat brains. Our results suggested that olanzapine induced inflammatory gene overexpression in peripheral adipose tissues, but not in the central nervous system.

### Olanzapine Inhibited the Glucose Utilization of Adipocytes by Inducing IR via NF-κB Activity Activation

**Figure 4:**
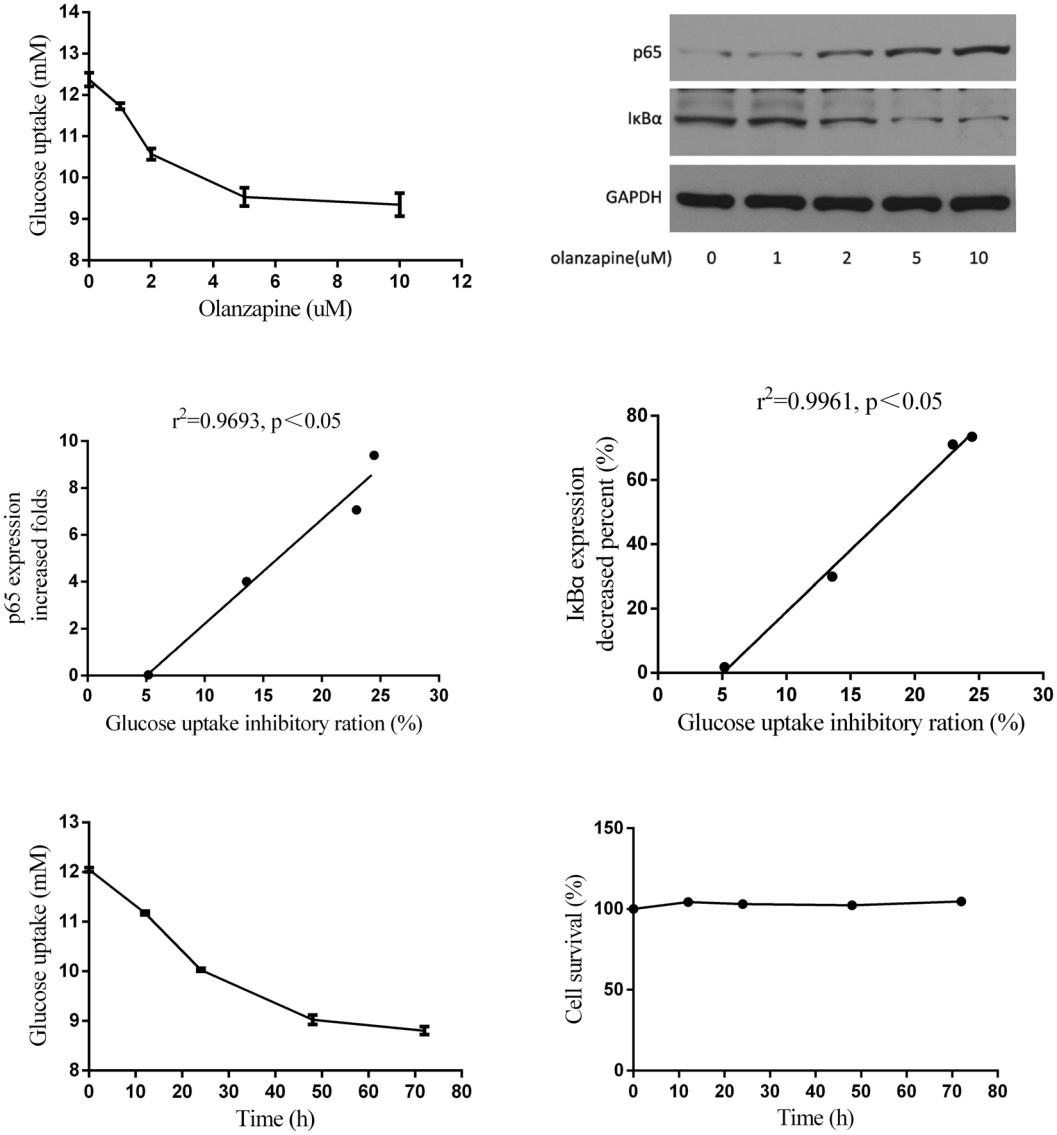
Olanzapine inhibited insulin-stimulated glucose utilization, induced NF-κB activity in a dose-dependent and time-dependent manner and did not inhibit cell survival. (**A**) Olanzapine dose-dependently inhibited insulin-stimulated glucose uptake and induced NF-κB activity. (**B**) Correlation between the decrease of insulin action and the increase of NF-κB activity. (**C**) Olanzapine inhibited insulin-stimulated glucose uptake over time and did not inhibit cell survival. Data in plot graphs are presented as mean ± SD. The Pearson correlation scores were calculated between p65 or IkBa expression and glucose uptake inhibitory ratio. One-way ANOVA statistical analyses, *post hoc t-test*, were performed for multiple comparisons. *p < 0.05.

NF-κB plays a key role in initiating and regulating inflammatory responses^1^. NF-κB typically exists as homodimers or heterodimers, among which the p50/p65 dimer is the most widely distributed and has important physiological functions^2^. To determine the potential pathway of olanzapine induced IR through NF-kB cascades in white fat tissue, we first incubated 3T3-L1 adipocytes with a series of olanzapine concentrations (0, 1, 2, 5, or 10 µM) for 48 h to investigate the effects of olanzapine on insulin sensitivity and NF-κB activity. As shown in the left panel of Fig. 4A, 1 µM olanzapine had no significant effect on the insulin-stimulated glucose uptake or the NF-κB (p65) activity compared with the control group (Fig. 4A right panel). Olanzapine significantly inhibited insulin-stimulated glucose uptake, increased the NF-κB activity and decreased the IκBα protein expression at 5 µM (Fig. 4A right panel). When the concentration was increased to 10 µM, the effect of olanzapine did not further increase (Fig. 4A). These results show that olanzapine dose-dependently inhibits the action of insulin and increases the activity of NF-κB, and there are good correlations between the decrease of insulin action and the increase of NF-κB activity or the decreased expression levels of IkBa (Fig. 4B).

To further investigate whether the effect of olanzapine is time dependent, we incubated 3T3-L1 adipocytes with 5 µM olanzapine for 0, 12, 24, 48 and 72 h. The results are shown in Fig. 4C; insulin-stimulated glucose uptake inhibited by olanzapine increased with time, which reached its maximum inhibition at 48 h. After incubation for 72 h, the inhibitory effect of olanzapine on insulin-stimulated glucose uptake did not increase significantly further. To rule out that long-term olanzapine treatment might disturb cell growth and survival, we conducted a CCK-8 experiment, which is an indicator of cell proliferation. The results showed that olanzapine 5 µM and 3T3-L1 adipocytes co-incubated for 48 h had no significant inhibitory effect on the survival of 3T3-L1 (Fig. 4C right panel). These results show that olanzapine inhibits insulin-stimulated glucose uptake by 3T3-L1 adipocytes over time, not because of the inhibition of cell survival.

### Olanzapine Stimulated NF-κB Activity through Dissociating NF-kB - IkBα Complex

**Figure 5:**
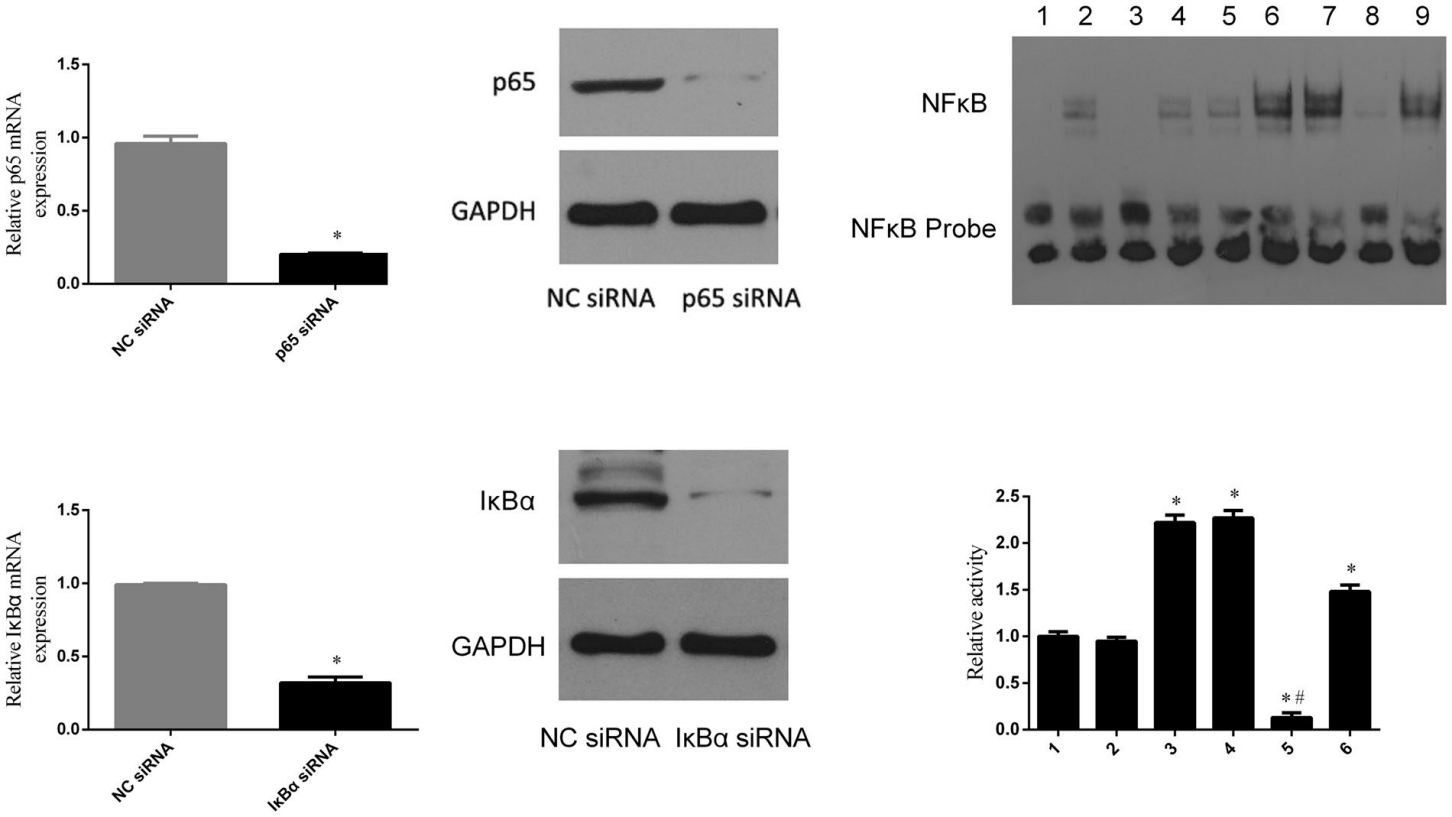
siRNA transfection significantly inhibited protein and mRNA expression, and interfered with NF-κB activity. (**A**) Effect of p65 siRNA transfection on p65 protein and mRNA expression. (**B**) Effect of IκBα siRNA transfection on IκBαprotein and mRNA expression. (**C**) Effect of olanzapine (upper 6#; lower 3#), IκBα siRNA (upper 9#; lower 6#) and co-incubation of olanzapine and p65 siRNA (upper 8#; lower 5#) on NF-κB activity. Upper panel: lane 1, Negative controal without cellular extracts; lane 2, Positive control with nuclear extract; lane 3, Competition assays with cold NF-κB oligonucleotides; lane 4, Vector control without Control siRNA and olanzapine; lane 5, Control siRNA transfected; lane 6, olanzapine treatment; lane 7, Control siRNA transfected and olanzapine treatment; lane 8, p65 siRNA transfected and olanzapine treatment; lane 9, IκBα siRNA transfected. Lower panel: lane 1, Vector control without Control siRNA and olanzapine; lane 2, Control siRNA transfected; lane 3, olanzapine treatment; lane 4, Control siRNA transfected and olanzapine treatment; lane 5, p65 siRNA transfected and olanzapine treatment; lane 6, IκBα siRNA transfected.One-way ANOVA statistical analyses, *post hoc t-test*, were performed for multiple comparisons. Data are expressed as mean ± SD. *p < 0.05 compared with control group; ^#^p < 0.05 compared with olanzapine group.

IκBα is a specific inhibitor of NF-κB^40^. At rest, IκBα binds to p65/p50 as an inactive complex in the cytoplasm; once activated, IκB kinase phosphorylates IκBα and dissociates it from NF-κB. Free NF-κB translocates into the nucleus and binds to DNA to activate the transcriptions of genes that encode inflammatory cytokines. To elucidate the effect of olanzapine on NF-κB activity, we specifically inhibited NF-κB using small interfering RNAs (p65 siRNA and IκBα siRNA). We compared the effects of olanzapine, p65 siRNA and IκBα siRNA on NF-κB activities. First, we transfected cells with IκBα siRNA and p65 siRNA, respectively (Supplemental Table 3). The mRNA and protein expressions of IκBα and p65 after transfection were detected by PCR and western blots. The results showed that p65siRNA could significantly knock-down p65 mRNA over 75% (Fig. 5A left panel) and substantially inhibited the protein expression to a 90% lower level (Fig. 5A right panel). Similarly, IκBα siRNA significantly inhibited the IκBα gene transcription close to 70% (Fig. 5B right panel), as well as the protein expression to 90% (Fig. 5B left panel).

To investigate the direct effect of olanzapine on NF-κB activities, NF-κB binding to DNA was performed by EMSA; NIH 3T3-L1 derived adipocytes were treated with olanzapine, IκBα siRNA, or along with p65 siRNA, and EMSA was performed using the nucleoproteins extracted from these cells. The results demonstrated that both olanzapine and IκBα siRNA significantly increased the binding of NF-κB to nuclear DNAs, which suggests that it could substantially activate cytokine production compared with the control group (Fig. 5C upper panel, lanes 4–7 and 9, and lower panel). As expected, the results showed that p65 siRNA significantly inhibited olanzapine-induced NF-κB activity (Fig. 5C upper panel, lane 8, and lower panel).

### IκBα Overexpression and p65 siRNA Rescued Insulin-stimulated Glucose Uptake and GLUT4 Membrane Transfer on Olanzapine-induced Adipocyte IR

**Figure 6:**
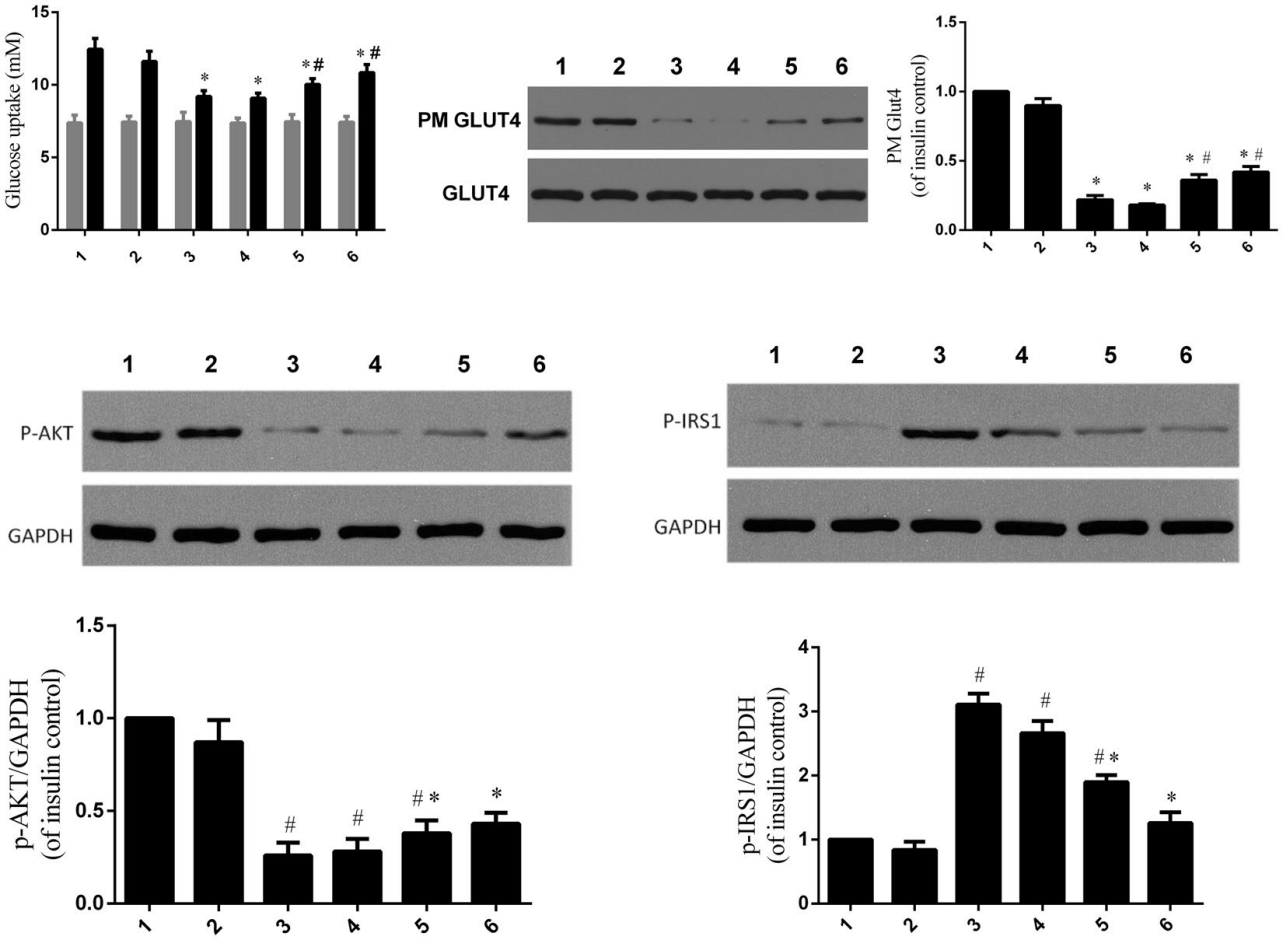
IκBα and p65 siRNA Rescued Insulin-stimulated Glucose Uptake and GLUT4 Membrane Translocation on Olanzapine-induced Adipocyte IR. (**A**) Effect of transfection reagents on insulin-stimulated glucose uptake (black bar) in olanzapine-treated adipocytes (olanzapine (lane 3), IκBα siRNA (lane 5), co-incubation of olanzapine and p65 siRNA (lane 6)). (**B**) Effect of transfection reagents (olanzapine (3# both left and right), IκBα siRNA (5#), co-incubation of olanzapine and p65 siRNA (6#) on insulin-stimulated GLUT4 translocation in olanzapine-treated adipocytes. (**C**) Effect of transfection reagents (olanzapine (3# both upper and lower panels), IκBα siRNA (5#), co-incubation of olanzapine and p65 siRNA (6#)) on p-IRS1 protein expression in olanzapine-treated adipocytes. (**D**) Effect of transfection reagents (olanzapine (3# both upper and lower panels), IκBα siRNA (5#), co-incubation of olanzapine and p65 siRNA (6#)) on p-AKT protein expression in olanzapine-treated adipocytes. lane 1, Control without Control siRNA and olanzapine; lane 2, Control siRNA transfected; lane 3, olanzapine treatment; lane 4, Control siRNA transfected and olanzapine treatment; lane 5, IκBα siRNA transfected; lane 6, p65 siRNA transefected and olanzapine treatment. One-way ANOVA statistical analyses, post hoc t-test, were performed for multiple comparisons of levels of plasma GLUT4, p-AKT, or p-IRS-1 among different treatments. Data in bar graphs are expressed as mean ± SD. *p < 0.05 compared with insulin group; ^#^p < 0.05 compared with olanzapine and insulin treatment group.

To elucidate the role of NF-κB in olanzapine-induced IR, we transfected p65 siRNA into 3T3-L1 adipocytes. We also transfected IκBα siRNA into 3T3-L1 adipocytes to determine whether increasing the NF-κB expression could cause IR. As shown in Fig. 6A, olanzapine significantly inhibited insulin-stimulated glucose uptake by as much as 30.8% after incubation for 48 h. However, 3T3-L1 adipocytes transfected with p65 siRNA followed by olanzapine treatment prevented olanzapine-induced decreases in insulin-stimulated glucose uptake. In addition, 3T3-L1 adipocytes transfected with IκBα siRNA prevented insulin-stimulated glucose uptake by 25.4% (p < 0.05) compared with the control group.

Insulin regulates glucose uptake by inducing GLUT4 from intracellular vesicles to the cell surface in adipose tissue. Thus, we examined the expression of GLUT4 in 3T3-L1 adipocytes. As shown in Fig. 6B, the total GLUT4 expression of 3T3-L1 adipocytes was not significantly different in the different groups. However, olanzapine treatment and IκBα siRNA transfection significantly reduced insulin-induced GLUT4 translocation as assessed by GLUT4 contents on the cell membrane (Fig. 6B right panel: olanzapine, 75.3%, p < 0.05; IκBα siRNA, 56.5%, p < 0.05). P65 siRNA transfection blocked the inhibitory effect of olanzapine on insulin-induced GLUT4 membrane translocation.

To further elucidate the role of NF-κB in olanzapine-induced IR, we subsequently examined the effects of olanzapine and siRNA treatment on insulin signaling proteins. As shown in Fig. 6C (upper and lower panels) and Fig. 6D (upper and lower panels), olanzapine significantly increased IRS-1 phosphorylation by 2-fold (p < 0.05) and decreased Akt phosphorylation by 68.8% (p < 0.05), whereas p65 siRNA treatment largely blocked the effect of olanzapine on IRS-1 and Akt. Compared with the control group, IκBα siRNA transfection significantly increased IRS-1 phosphorylation by 1-fold (p < 0.05) and decreased Akt phosphorylation by 37.5% (p < 0.05).

## Discussion

**Figure 7:**
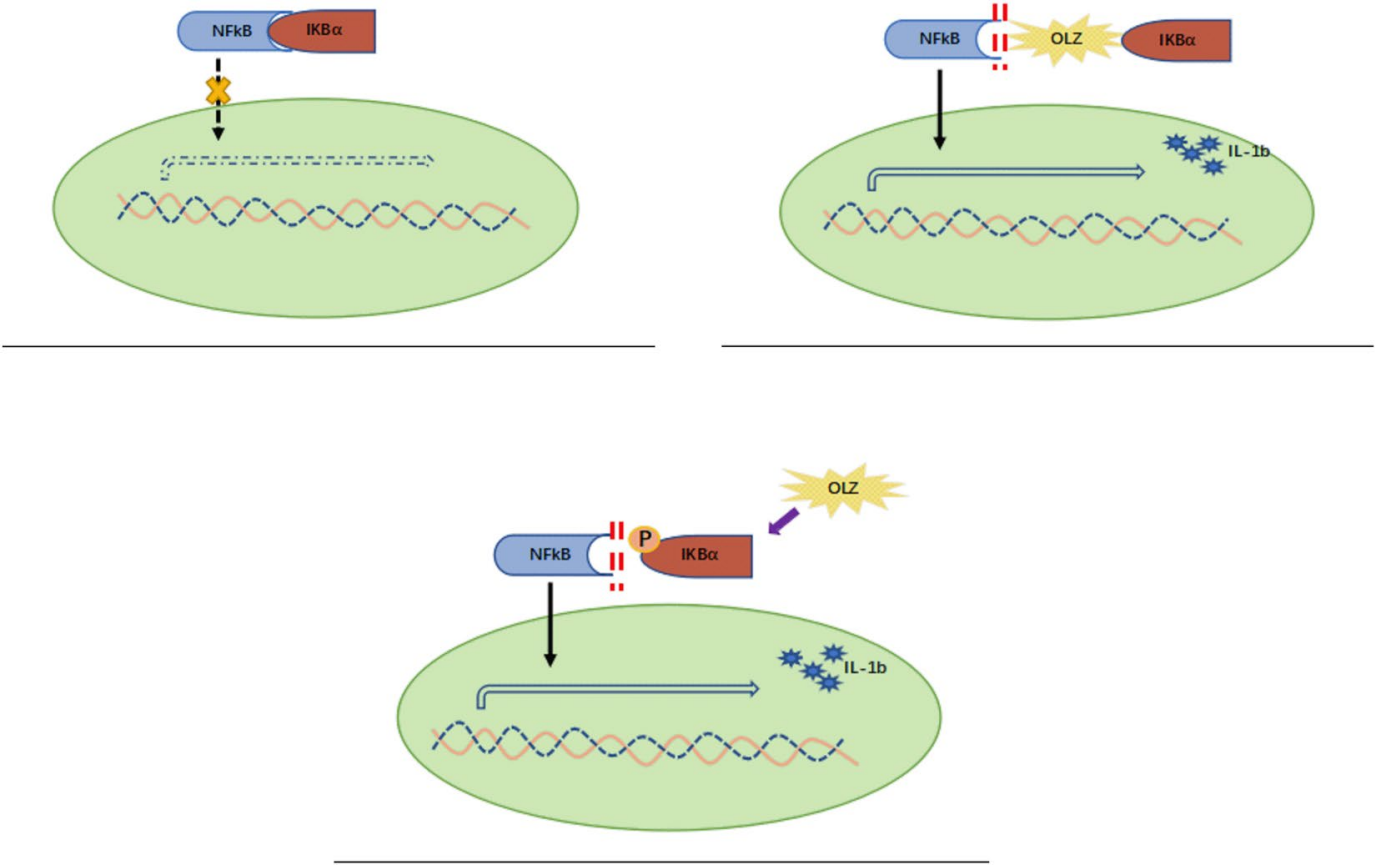
The schematic diagram shows possibility of the NF-kB-IkBa complex contributes to olanzapine-induce IR. (**A**) IkBα binds to NF-Kb inhibiting NF-Kb transduction into adipose nuclei in physiological conditions. (**B**) Olanzapine may physically block the formation of NF-Kb-IKbα complex, leading to activate inflammatory reactions in adipose tissue. (**C**) Olanzapine may activate phosphorylation of IkBα and then free NF-Kb from their complex, leading to activate inflammatory reactions in adipose tissue.

Olanzapine has been widely prescribed as a maintenance treatment for severe schizophrenia illness. Olanzapine is also widely used for the treatment of MDD and bipolar disorders; however, it is facing increasing noncompliance due to its reverse metabolic symptoms and insulin-resistance (IR), which are strong risk factors for type-2 diabetes. To uncover olanzapine induced IR, we investigated the effects of inflammatory cytokines in schizophrenia patients and demonstrated that inflammation is a key player of olanzapine induced IR using rodent mouse and rat models. We further explored the effects of olanzapine on NF-κB and insulin activities based on the 3T3-L1 adipocyte system. Our results indicated that olanzapine ignites inflammatory responses in peripheral adipose tissues by dissociating the NF-κB – IkBα complex to free NF-kB, which transduces into adipocyte nuclei to prompt the expression of inflammatory cytokines (Fig. 7). Furthermore, our results imply that activated NF-kB might induce the serine phosphorylation of insulin receptor substrate-1 (IRS-1) to downphosphorylate Akt and subsequently inhibits the translocation of GLUT4 to the adipose cell membrane and inhibits insulin utilization. We have also confirmed that the inhibition of NF-κB could significantly diminish IR and alleviate the inhibitory effect of olanzapine on insulin-stimulated glucose uptake in the adipocyte system. More interestingly, our current study, for the first time, showed that olanzapine induces no inflammatory response in the central nervous system, although it initiates strong inflammatory cytokine reactions in peripheral adipose tissue to induce IR.

Many cohort studies have shown that approximately 30% of diabetes or IR were induced by antipsychotics^41,42^. Schizophrenia patients from our clinical olanzapine long-term treatments showed a similar IR incidence and had significantly increased plasma glucose levels and HOMA-IR index, with a clear IR state that worsened glucose control in the OR patients compared to the OS or normal control subjects. Our clinical data proved that chronic olanzapine treatment induces IR and increases the risk of hyperglycemia, consistent with previous studies^43,44^. To clarify whether inflammation is a common player in IR, we examined the plasma inflammatory cytokine levels in the 14 OR and 14 OS patients and found that TNF-α, IL-6, IL-8 and IL-1β were significantly upregulated in the OR group compared with the OS group; these findings indicate an inflammatory response as an authentic indicator of IR (Table 1 and Fig. 1). Fountaine *et al*.^45^ also found that serum inflammatory factors, the levels of TNF-α and PAI-1, were significantly elevated in healthy male subjects after long-term application of olanzapine (5–10 mg/d)^45^. Major recent studies have suggested that inflammation could also be a mechanism for inducing IR^46,47^. However, for the first time, our results indicated that long-term olanzapine may induce hyperglycemia and IR by activating an inflammatory response in the peripheral adipose system.

We chose female rodent mice and rats to mimic the model of clinical long-term olanzapine treatment. Peripheral adipose tissue was comprehensively examined for pro-inflammatory factors, and our results clearly demonstrated that the plasma glucose and insulin levels were significantly increased in these established rodent models after 8 weeks of olanzapine treatment. The HOMA-IR index and the AUC in the OGTT were also significantly increased, which indicates that long-term olanzapine induced IR in the adipose system. Moreover, there was no significant difference in the body weight between the non-IR and IR animal groups, which indicates that long-term olanzapine treatments induced IR independent of weight gain in these rodent models (Supplemental Table 2 and Fig. 2). These data are consistent with recent clinical reports that weight-gain might not be a clue of olanzapine induced IR^43^.

NF-κB is a key regulator of the transcription of inflammatory cytokines. At rest, NF-κB binds to IκBα in the cytoplasm. After stimulation, phosphorylated IκBα degrades and dissociates with NF-κB. After dissociation, NF-κB enters the nucleus and binds to DNA, inducing the transcription of inflammatory cytokines^32^. In the current study, 3T3-L1 derived adipocytes treated with olanzapine showed that olanzapine concentration-dependently inhibited insulin-stimulated glucose uptake in the 1–5 µM range, consistent with the findings of Vestri *et al*.^44^. Our results also demonstrated the effect of olanzapine on increasing insulin and NF-κB in a time-dependent manner. Our results have shown that olanzapine significantly decreased the IκBα expression and increased the NF-κB expression and activity in 3T3-L1 adipocytes. Consistent with our findings, Sarvari *et al*. demonstrated that olanzapine significantly induced the expression of NF-κB and its target genes, such as TNF-α, IL-1β and IL-8, in human adipocytes *in vitro*^48^. These observations from cellular and molecular levels further confirmed that the IκBα- NF-κB complex pathway plays a pivotal role in olanzapine induced IR. These observations from cellular and molecular levels further confirmed that the IκBα- NF-κB complex pathway plays a pivotal role in olanzapine induced IR. Whether olanzapine physically blocks IkBa or activates IkBa to release free NF-kB leading to IR warrants additional studies.

To further confirm the IκBα- NF-κB complex pathway mechanism in IR, transfected 3T3-L1 adipocytes with p65 siRNA did show significantly downregulated NF-κB expression to relieve the IR status. The results showed that olanzapine and IκBα siRNA significantly induced NF-κB activity, inhibited insulin-stimulated glucose uptake and GLUT4 membrane translocation, and inhibited insulin signaling. The p65 siRNA can inhibit the activity of NF-κB to partially block the effect of olanzapine on NF-κB and IR. However, compared with IκBα siRNA transfection, olanzapine induced a more significant inhibition on insulin activity in the current study. Co-incubation with p65 siRNA did not fully rescue insulin-stimulated glucose uptake. These observations suggest that IR by olanzapine may have more signaling pathways other than NF-κB-dependent pathways.

These results indicate that long-term olanzapine-induced IR is related to the upregulation of inflammatory cytokines and inflammation, which suggests that NF-κB may be a potential target for the prevention and alleviation of olanzapine-induced IR. The most important finding of the current study is the first-time demonstration that olanzapine induced the expressions of inflammatory cytokines in the peripheral but not in the central nervous system. Although the increased adipose IL-6 levels were consistent with the findings from the Calevro^29^ group, the same animals showed cortical region-specific changes in the microglia density and activation state^49^, which indicates further investigation is required to link the microglial activation between peripheral and central cytokines after chronic olanzapine treatment. However, this current work could imply a new era to illuminate the mechanism by which olanzapine or other antipsychotic drugs may have differential effects on the central nervous system and peripheral adipose system.

## Methods

### Patient Recruitment Process and Sample Collection

This study recruited schizophrenia patients with long-term administration of olanzapine, whom were diagnosed positive, negative and cognitive impairment or emotional syndrome, according to the PNASS factor scores. Inclusion criteria: (1) age 18–60 years old;(2) no infectious disease, no chronic cardiovascular, digestive tract, endocrine, immune system and respiratory diseases within two weeks before blood sampling; (3) no family history of diabetes and no major liver or kidney disease; no bad habits, such as addiction and alcohol abuse; (4) nonpregnancy or lactation; (5) treatment with other mood stabilizers or other atypical antipsychotics (with olanzapine as the primary drug), but no antihypertensive drugs or typical antipsychotics; (6) informed consent; (7) patients treated mainly with olanzapine over 2 months and without hypoglycemic drugs. This clinical study was approved by the Tongji Medical College Ethics Committee of Huazhong University of Science and Technology (HUST).

We called clinical patients with long-term administration of olanzapine from different hospitals to conduct this study, which included measuring the body weight, fasting blood glucose, and insulin levels and calculating the insulin resistance index (HOMA-IR index). In this study, we mainly relied on experienced clinicians to determine the grouping of patients. In clinical practice, they assessed the patient’s metabolic status and insulin sensitivity based on changes in body weight and fasting blood glucose after long-term olanzapine treatment. Based on the HOMA-IR index values, the patients were divided into the OS group (the insulin resistance index has similar variations as the average levels in normal subjects, data not shown) and the OR group (the insulin resistance index is significantly higher than that of the average levels in normal subjects).

All experiments were performed according to the guidelines and regulations under HUST. The demographic and clinical details of the samples are provided in Table 1.

After overnight fasting, 10-ml blood samples were obtained from the patients and placed in a test tube that contained EDTAK2 anticoagulant. Following centrifugation at 3000 rpm for 10 min, the plasma was stored at −80 °C for later use.

### Animals, Drug Treatment and Examination of Inflammatory Factors

Adult female Balb/c mice and Sprague Dawley rats (purchased from Beijing Huafu Kang Biotechnology Co., Ltd., animal license number: SCXK (Beijing) 2014–0004) were group-housed and maintained in the SPF animal room of the Animal Experiment Center of HUST with a 12-hour light/dark cycle. The animal use and procedures were in accordance with the HUST Animal Ethics Committee regulations and requirements. All experiments were performed under HUST animal guidelines and regulations.

The appropriate amount of olanzapine (TCI, Japan) was dissolved with 0.1 M hydrochloric acid solution, with 1 M sodium hydroxide solution to adjust the pH to 6.0. Olanzapine was intraperitoneally injected with dose justification as described and discussed in Wu, *et al*.^50^. Briefly, the injection doses were 10 mg/kg, injection volume of 10 ml/kg, with 0.1 M hydrochloric acid as the control. One week after the rodent animals were adaptively fed, they were randomly divided into two groups: the olanzapine treatment group and the control group. The olanzapine and control solvent were orally administered at 8:30 a.m. to 9 a.m. daily for 8 weeks.

After 8 weeks of administration, the body weight, fasting blood glucose and insulin levels were measured, and the Homeostasis model assessment insulin resistance (HOMA-IR) was calculated as shown in Supplemental Table S2. HOMA-IR = fasting blood glucose (mmol/L) * fasting insulin (mU/L) /22.5. The oral glucose tolerance test (OGTT) was performed after the end of the administration, and the area under the blood glucose curve was calculated. Orbital blood samples were obtained in heparin-added EP tubes, plasma was collected, and white adipose tissue was carefully separated. The prefrontal cortex of the rat brains from the OS and OR groups were dissected and immediately stored at −80 °C for later use.

Blood glucose was measured by a blood glucose detector. Insulin and inflammatory cytokines (TNF-α, IL-6, IL-8 and IL-1β) were measured using ELISA Kits (Neobioscience, China).

### Adipocyte Cell Line and Differentiation Process

3T3-L1 fibroblasts (Shanghai Meixuan Biotechnology Co., Ltd.) were cultured in DMEM medium (which contained 4 mM L-glutamine, 4.5 g/L glucose and 10% fetal bovine serum) in a 5% CO_2_ incubator. After 2 days of fusion, the cells were stimulated for fat synthesis in DMEM medium that contained 25 mmol/L glucose, 0.5 mmol/L isobutylmethylxanthine, 1 mmol/L dexamethasone, 10 mg/ml insulin and 10% fetal bovine serum. After 3 days, the cells were changed using DMEM medium with 10 mg/ml insulin and 10% FBS. The medium was changed every 2 days during 10–14 day experiment, when 90–95% cells showed adipocyte phenotype.

### Olanzapine Treatment of Adipocytes

After 3T3-L1 adipocytes (or transfected cells) were incubated with serum-free, high glucose, DMEM medium and olanzapine at the indicated concentrations were applied for the indicated time, the samples were incubated with 100 nM insulin or blank control solution without insulin for 30 min, and the supernatants were collected. The GOD-POD kit (Nanjing Jiancheng Bioengineering Institute, China) was used to determine the glucose content in the supernatants. The initial glucose content in the medium was subtracted from the glucose content in the supernatant to obtain the glucose content absorbed by the cells. The control cells were incubated with solvent only.

### Examination of Influence on Adipocyte Growth

After the 3T3-L1 adipocytes were treated with olanzapine under the specified conditions, 10 µL CCK-8 solution was added, and the cells were incubated in an incubator at 37 °C for 2 h. The absorbance at 450 nm was measured using a microplate reader to calculate the effect of olanzapine on cell growth.

### Gene Expression and Knock-down

IκBα siRNA and P50/p65 siRNA were synthesized by Shanghai Gemma (Ribobio, China). Briefly, prior to transfection, cells were harvested by trypsinization and collected, and the cell density was adjusted to 2 × 10^5^/ml. Corresponding siRNAs were transfected into 3T3-L1 adipocytes using Lipofectamine rTM2000. The positive and vector siRNA were used to assess the transfection and knock-down efficiency. The levels of mRNA and protein expression of the target genes were detected after 48 hours.

### Western Blotting Analyses

All cell lysates were collected via low-speed centrifugation. Briefly, cells were incubated ice-cooled lysis buffer for 30 minutes, vortexed and extracted the protein via centrifugation; the protein concentration was determined using the BCA method. SDS-PAGE electrophoresis and western blotting procedures were performed. Inflammation activates serine but not threonine phosphorylation of the insulin receptor substrate (IRS-1). Thus, the specific rabbit anti serine p-IRS1 antibody was purchased and applied in the present study, Cat:#2385, CST, USA, 1:500; the other corresponding protein primary antibodies used in this study were as follows: p65, (Cat:#8242, CST, USA, 1:2000); IκBα, (Cat:ab32518, Abcam, England, 1:2000); GLUT4, (Cat:ab33780, Abcam, England, 1:1000); p-AKT, (Cat:#4060, CST, USA, 1:2000); and GAPDH, (Cat:ab37168, Abcam, England, 1:10000). All primary antibodies were raised from rabbits; thus, a secondary antibody (HRP-Goat anti Rabbit, Cat:AS1107, ASPEN, USA, 1:10000) was used for each western blot analysis. Gel imager detection was performed with an X-ray film reader (Kodak, Japan). A cell membrane GLUT4 assay using a membrane protein extraction kit was used to determine the GLUT4 protein expression.

### Quantitative Real-Time PCR

Approximately 100 mg of mouse adipose tissue was obtained and extracted by Trizol extraction (15596–026, Invitrogen™). The extracted RNA was added to the reverse transcription reaction system for reverse transcription. The primers for TNF-α were 5′-TCCCCAAAGGGATGAGAAGTT-3′ and 5′-GAGGAGGTTGACTTTCTCCTGG-3′. The primers for IL-6 were 5′-CTGGGAAATCGTGGAAATGAG-3′ and 5′-AAGGACTCTGGCTTTGTCTTTCT-3′. The primers for IL-1β were 5′-GGGCCTCAAAGGAAAGAATCT-3′ and 5′-GAGGTGCTGATGTACCAGTTGG-3′. The primers for IL-8 were 5′-GGCCCAATTACTAACAGGTTCC-3′ and 5′-TGACTTCACTGGAGTCCCGTAG-3′. The primers for GAPDH were 5′-TGAAGGGTGGAGCCAAAAG-3′ and 5′-AGTCTTCTGGGTGGCAGTGAT-3′. Real-time PCR was performed on a StepOne™ Real-Time PCR instrument using the SYBR® Premix Ex Taq™ kit (RR047A, TaKaRa, Japan). The calculation formula was as follows: ΔCt = Ct value of the target gene - Ct value of the reference gene; ΔΔCt = ΔCt of control group - ΔCt of the experimental group; the relative expression value of the target gene in the sample of the experimental group is 2^ΔΔCt^.

### Electrophoretic Mobility Shift Assay

Following the cell intervention experiment, the nucleoprotein was extracted, and the protein concentration was determined with the purchased kit. Gel was prepared. After the gel was completely solidified, it was electrophoresed for 1 h at 100 v. After the pre-electrophoresis was completed, the precooled electrophoresis buffer was replaced, and 5 µl of 5x sample buffer was added to the sample mixture. Electrophoresis was then immediately performed at 150 v for 30–45 minutes. The positively charged nylon membrane was placed in a 0.5 x TBE for 10 minutes. After the electrophoresis was completed, the entire block of the sample was removed and transferred. After the membrane was completed, the membrane was marked and cross-linked under UV for 10 min. The membrane was blocked with blocking solution for 15 minutes. After being diluted 300-fold with blocking solution, the antibody was fully reacted with the membrane for 15 minutes. The membrane was subsequently washed and balanced. The image to the protein side of the membrane was taken. The film was scanned and analyzed with the AlphaEaseFC system. The oligos were AGTTGAGGGGACTTTCCCAGGC(5′−3′) and TCAACTCCCCTGAAAGGGTCCG(5′−3′).

### Statistical Analyses

The PANSS scaling was used to measure the symptom severity of the patients with schizophrenia. The minimum scores for the positive, negative and general scales are 7, 7, and 16 and are used for the matched controls. Student’s t test was used to compare two groups for continuous variables. A Chi-square test was used to compare two groups for binary variables.

SPSS19.0 (SPSS Inc., IBM, USA) was used to process all animal and cell experimental data as mean ± SD. Analysis of variance (ANOVA) was used to compare multiple groups, and the least significant difference (LSD) was used for comparisons among groups. A logistic regression model was used for variables of each inflammatory factor with covariates of the HOMA-IR index. Pearson correlation scores were calculated for every inflammatory factor and the normalized HOMA-IR index values.

Statistical significance was set at p < 0.05. All experiments were repeated at least 3 times.

### Ethical approval and informed consent

The use of all human samples and blood tissues in this study complied with the guidelines and regulations of the Tongji Medical College Ethics Committee of Huazhong University of Science and Technology (HUST). All experiments on human samples were approved by the Tongji Medical College Ethics Committee of HUST.

All experiments on rodents were approved by the HUST Animal Ethics Committee and were performed under the HUST animal guidelines and regulations.

## Supplementary information

supplemental materials

## Data Availability

All raw data, charts, and figures in this manuscript are available as electronic files uploaded on internet resources of *scientific reports*.
