# Supplementary material for: Chronic olanzapine administration causes metabolic syndrome through inflammatory cytokines in rodent models of insulin resistance
Source: Sci Rep. 2019 Feb 7;9:1582. doi: 10.1038/s41598-018-36930-y (PMC6367387; doi:10.1038/s41598-018-36930-y)
Supplement: Supplementary file 1 — supplemental materials [file 41598_2018_36930_MOESM1_ESM.docx]

**Chronic olanzapine administration causes metabolic syndrome through inflammatory cytokines in rodent models of insulin resistance**

Huqun Li^1&^, Shiyong Peng^2&^*, Shihong Li^1^, Shouqing Liu^2^, Yifan Lv^2^, Ni Yang^1^, Liangyu Yu^1^, Ya-Hui Deng^1^, Zhongjian Zhang^2,3^, Maosheng Fang^4^, Yunxiang Huo^5^, Ying Chen^5^, Taohua Sun^6^, Weiyong Li^1*^

**Addresses:**

1，Department of Pharmacy, Central Hospital of Wuhan, Wuhan, PR China 430014; Department of Pharmacy, Union Hospital Affiliated to Tongji Medical College, Huazhong University of Science and Technology, Wuhan, PR China 430000

2，Institute of Psychiatry and Neuroscience, Xin Xiang Medical University, Xinxiang, Henan, China 435000

3，Section on Developmental Genetics, PDEGEN, NICHD, Bethesda, Maryland 20892, USA

4, Wuhan mental health center, Wuhan, PR China 430019

5, Wuhan Youfu Hospital, Wuhan, PR China 430050

6, Qingdao Municipal Hospital, Qingdao, PR China 266011

^&^Contributed equally.

*To whom all correspondence should be addressed: Tel: 86-027-85726063; Tel: 86-373-3831324; Email: [2621239868@qq.com](mailto:2621239868@qq.com); Email: [sampeng@XXMU.edu.cn](mailto:sampeng@XXMU.edu.cn)

Supplemental Table 1 Body weight, fasting glucose, insulin and HOMA-IR index in rodent model.

| Parameters | mouse | | rat | |
| --- | --- | --- | --- | --- |
|  | CG group | OL group | BL group | IR group |
| age | 8 week | | 8 week | |
| Weight (g) | 21.44±0.19 | 21.88±0.04 | 271.93±21.82 | 323.10±17.91^*^ |
| Fasting glucose (mmol/L) | 3.2±0.2 | 11.2±0.4* | 4.48±0.38 | 5.23±0.93^*^ |
| Fasting insulin (mU/L) | 23.8±0.4 | 33.4±0.3* | 19.41±6.84 | 28.30±7.59^*^ |
| HOMA-IR index | 3.40±0.15 | 17.26±0.12* | 3.89±1.46 | 6.69±2.22^*^ |
| AUC (mmol·min/L) | 885.8±17.7 | 1616.2±7.0* | 780.75±84.87 | 839.08±101.53^*^ |

CG, the control group in mouse; OL, the olanzapine treatment group in mouse; BL,,the control group in rat; IR, the olanzapine treatment group in rat; HOMA-IR index=fasting blood glucose (mmol / L) * fasting insulin (mU / L) /22.5. AUC: area under curve. *: p <.05 compared with mice or rats in control group.

A B







C D







Supplemental figure 1 Chronic olanzapine treatment altered inflammatory cytokines in mice, and inflammatory cytokines were correlated to IR within OL groups. (A) Correlation between TNF-α and HOMA-IR index. (B) Correlation between IL-6 and HOMA-IR index. (C) Correlation between IL-1β and HOMA-IR index. (D) Correlation between IL-8 and HOMA-IR index. n=10 mice per group. Data are presented as mean ± SD. *: p<.05.

Supplemental table 2. Regression coefficients and their 95% confidence intervals from linear regression analysis of inflammatory factors and parameters in patients.

|  | TNF-α | | | | IL-6 | | | |
| --- | --- | --- | --- | --- | --- | --- | --- | --- |
|  | B | p | 95%CI | | B | p | 95%CI | |
| Age | 0.024 | 0.799 | -0.165 | 0.213 | 0.019 | 0.807 | -0.145 | 0.184 |
| Gender | -1.304 | 0.520 | -5.436 | 2.827 | -0.371 | 0.823 | -3.798 | 3.056 |
| Weight | 0.046 | 0.552 | -0.112 | 0.204 | 0.008 | 0.896 | -0.121 | 0.137 |
| IR index | 9.161 | 0.000 | 4.576 | 13.746 | 6.702 | 0.002 | 2.920 | 10.485 |
|  | IL-1β | | | | IL-8 | | | |
| Age | 0.036 | 0.661 | -0.132 | 0.203 | 0.010 | 0.917 | -0.180 | 0.199 |
| Gender | -0.511 | 0.762 | -4.003 | 2.981 | -1.011 | 0.597 | -4.958 | 2.937 |
| Weight | 0.008 | 0.902 | -0.124 | 0.139 | 0.008 | 0.907 | -0.140 | 0.157 |
| IR index | 5.751 | 0.006 | 1.898 | 9.605 | 7.485 | 0.002 | 3.129 | 11.842 |

B=unstandardized regression coefficient; CI=Confidence Interval.

Supplemental table 3. Regression coefficients and their 95% confidence intervals from linear regression analysis of inflammatory factors and parameters in mice.

|  | TNF-α | | | | IL-6 | | | |
| --- | --- | --- | --- | --- | --- | --- | --- | --- |
|  | B | p | 95%CI | | B | p | 95%CI | |
| Weight | -6.720 | 0.173 | -16.688 | 3.249 | -2.178 | 0.619 | -11.245 | 6.889 |
| IR index | 7.143 | 0.000 | 6.131 | 8.155 | 4.540 | 0.000 | 3.620 | 5.461 |
|  | IL-1β | | | | IL-8 | | | |
| Weight | .804 | 0.627 | -2.627 | 4.236 | 0.370 | 0.880 | -4.737 | 5.477 |
| IR index | 3.313 | 0.000 | 2.965 | 3.661 | 4.902 | 0.000 | 4.383 | 5.420 |

B=unstandardized regression coefficient; CI=Confidence Interval
